# A small secreted protein serves as a plant-derived effector mediating symbiosis between *Populus* and *Laccaria bicolor*

**DOI:** 10.1093/hr/uhae232

**Published:** 2024-08-09

**Authors:** Yang Liu, Feng Zhang, Amith R Devireddy, Raphael A Ployet, Tomás A Rush, Haiwei Lu, Md Mahmudul Hassan, Guoliang Yuan, Ruchika Rajput, Md Torikul Islam, Rekha Agrawal, Paul E Abraham, Jin-Gui Chen, Wellington Muchero, Francis Martin, Claire Veneault-Fourrey, Xiaohan Yang

**Affiliations:** Biosciences Division, Oak Ridge National Laboratory, 1 Bethel Valley Road, Oak Ridge, TN 37831, USA; State Key Laboratory of Herbage Improvement and Grassland Agro-ecosystems, College of Ecology, Lanzhou University, 222 Tianshui S Rd, Chengguan District, Lanzhou, Gansu 730000, China; UMR 1136 Interactions Arbres-Microorganismes, Centre INRAE Grand Est-Nancy, INRAE, Université de Lorraine, Champenoux 54280, France; Biosciences Division, Oak Ridge National Laboratory, 1 Bethel Valley Road, Oak Ridge, TN 37831, USA; Biosciences Division, Oak Ridge National Laboratory, 1 Bethel Valley Road, Oak Ridge, TN 37831, USA; Biosciences Division, Oak Ridge National Laboratory, 1 Bethel Valley Road, Oak Ridge, TN 37831, USA; Biosciences Division, Oak Ridge National Laboratory, 1 Bethel Valley Road, Oak Ridge, TN 37831, USA; Department of Academic Education, Central Community College – Hastings; Hastings, NE 68901, USA; Biosciences Division, Oak Ridge National Laboratory, 1 Bethel Valley Road, Oak Ridge, TN 37831, USA; Department of Genetics and Plant Breeding, Patuakhali Science and Technology University, Dumki, Patuakhali, 8602, Bangladesh; Biosciences Division, Oak Ridge National Laboratory, 1 Bethel Valley Road, Oak Ridge, TN 37831, USA; Chemical and Biological Process Development Group, Pacific Northwest National Laboratory, 902 Battelle Boulevard, Richland, WA 99352, USA; Biosciences Division, Oak Ridge National Laboratory, 1 Bethel Valley Road, Oak Ridge, TN 37831, USA; Biosciences Division, Oak Ridge National Laboratory, 1 Bethel Valley Road, Oak Ridge, TN 37831, USA; Biosciences Division, Oak Ridge National Laboratory, 1 Bethel Valley Road, Oak Ridge, TN 37831, USA; Biosciences Division, Oak Ridge National Laboratory, 1 Bethel Valley Road, Oak Ridge, TN 37831, USA; Biosciences Division, Oak Ridge National Laboratory, 1 Bethel Valley Road, Oak Ridge, TN 37831, USA; Biosciences Division, Oak Ridge National Laboratory, 1 Bethel Valley Road, Oak Ridge, TN 37831, USA; UMR 1136 Interactions Arbres-Microorganismes, Centre INRAE Grand Est-Nancy, INRAE, Université de Lorraine, Champenoux 54280, France; UMR 1136 Interactions Arbres-Microorganismes, Centre INRAE Grand Est-Nancy, INRAE, Université de Lorraine, Champenoux 54280, France; Biosciences Division, Oak Ridge National Laboratory, 1 Bethel Valley Road, Oak Ridge, TN 37831, USA

Dear Editor,

Beneficial symbiotic fungi colonize plant tissues, delivering crucial ecosystem services such as carbon sequestration and plant fertilization [1]. Specifically, trees that form a nutrient-acquiring symbiosis with mutualistic ectomycorrhizal (ECM) fungi gain advantages from these associations by experiencing enhanced growth rates and increased resilience to both biotic and abiotic stresses [2, 3]. Despite the vital role ECM fungi play in the nutrition and well-being of trees, identifying key regulators participating in the molecular communication between plant and fungal cells is still in its early stages [4]. The mutualistic relationship between *Laccaria bicolor* and *Populus* spp. has been utilized as a model system for investigating ECM symbiosis at the molecular level. It has been demonstrated that the fungus *L. bicolor* secretes mycorrhiza-induced small secreted proteins (MiSSPs) required for ECM development [5]. Meanwhile, we have previously shown that *Populus trichocarpa* small, secreted proteins (PtSSPs) are highly induced during mutualistic symbiosis and some of them can enter, via *in vitro* feeding, *L. bicolor* hyphae affecting their growth and morphology [6]*.* However, the exact role and mode of action of PtSSPs in mutualistic symbiosis remain unknown. Because previous study showed that PtSSP1 is taking up by fungal cell and then localize in fungal cells [6], we decide to dig further on its putative role in fungal cells and also by overexpressing it in poplar because it is not technically possible yet to overexpress poplar protein in *Laccaria bicolor* hyphae. Here, we further characterized the function of PtSSP1(Potri.009G063200) in ectomycorrhization, which accumulates in the nucleus of *L. bicolor* in an *in vitro* feeding experiment [6]. Our results provide new knowledge for the genetic engineering of plants to control associated microbes.

To investigate the function of PtSSP1 in ectomycorrhization, we constitutively overexpressed *PtSSP1* in the hybrid poplar clone INRA 717-1B4 (*P. tremula × P. alba* clone INRA 717-1B4) using the CAMV35S promoter and investigated ectomycorrhiza development with *L. bicolor*. Three overexpression lines (251–1, 251–2, 251–3) were confirmed by visualization of fluorescence from the reporter GFPuv under UV light [7] and RT-qPCR analysis of transgene expression (Fig. 1A and B). For 3 weeks, we co-cultured established poplar roots with *L. bicolor* and investigated the ECM root tip formation. We found that overexpression of *PtSSP1* significantly increased the ECM root tip formation in two overexpression lines (251–2, 251–3) when inoculated with *L. bicolor* (*P* < 0.05, Fig. 1C and D). Specifically, the average percentage of ECM root tips in these two lines was approximately 72% after 3 weeks of co-culture with *L. bicolor*, while the average percentage of colonized root tips in empty vector controls was 59.2% (Fig. 1D).

**Figure 1:**
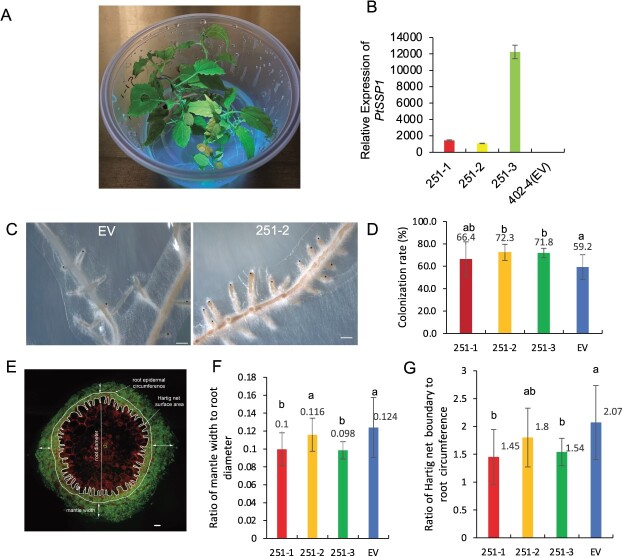
Impact of *PtSSP1*-overexpression on ectomycorrhiza (ECM) development with *Laccaria bicolor* and root development (A) Visualization of transgenic poplar plants under UV light. (B) Relative expression level of *PtSSP1* in three transgenic lines and one empty vector control line. EV indicates empty vector. Error bars indicate SD. Sample size is 3. (C) Representative stereomicroscope images of the empty vector control (EV) and *PtSSP1* overexpression (OE) plant (251–2) roots co-cultured with *L. bicolor* for three weeks. Black asterisks indicate the ECM root tips. Scale bar = 0.2 mm. (D) Percentage of ECM root tip formation in the *PtSSP1* OE lines and EV control plants. Error bars represent the standard deviation (SD). *n* = 5–9. The data were statistically analyzed by *t*-test to assign the significance groups a and b (*P* <0.05). (E) Representative transverse cross sections of *Populus* roots co-cultured with *L. bicolor* for 3 weeks. Four different measurements, including mantle width, root diameter, Hartig net boundary, and root circumference as labeled in the picture, were acquired for each image using ImageJ. Scale bar = 20 μm. (F) Mantle width to root diameter ratio as a proxy for external fungal growth. Error bars indicate SD. *n* = 10–18 per line. The data were statistically analyzed by *t*-test to assign the significance groups a and b (*P* <0.05). (G) Hartig net boundary to root circumference ratio as a proxy for *in planta* fungal growth. Error bars indicate SD. *n* = 10–18 per line. The data were statistically analyzed by *t*-test to assign the significance groups a and b (*P* <0.05). (H) Number of root tips. Error bars indicate SD. *n* = 3–5. The data were statistically analyzed by one-way ANOVA with Tukey pairwise comparison to assign significance groups a and b (*P* < 0.05). (I) Total root length. Error bars indicate SD. *n* = 3–5. The data were statistically analyzed by one-way ANOVA with Tukey pairwise comparison to assign significance groups a and b (*P* < 0.05). (J) Root volume. Error bars indicate SD. *n* = 3–5. The data were statistically analyzed by one-way ANOVA with Tukey pairwise comparison to assign significance groups a and b (P < 0.05). (K) Root surface area. Error bars indicate SD. *n* = 3–5. The data were statistically analyzed by one-way ANOVA with Tukey pairwise comparison to assign significance groups a and b (*P* < 0.05). (L–N) PtSSP1 interacts with the LbGAL4-like protein *in vitro* and in plant cells. (L) Confirmation of the interaction of PtSSP1 with the LbGAL4-like protein using the Yeast-two-hybrid assay. Yeast harboring bait (BD) and prey (AD) plasmids were grown on synthetic complete medium (SC)-Leu (L)-Trp (W)-Ura (U) (left), and SC-Leu (L)-Trp (W) (right) agar media at 30°C for 3 days before cell growth was evaluated. The combination of Krve1/RalGDS-wt was used as a positive control, while the combination of Krve1/RalGDS-m2 was a negative control. (M) Bimolecular fluorescence complementation (BiFC) assay in infiltrated *Nicotiana benthamiana* leaves, indicating an *in planta* interaction between PtSSP1 and LbGAL4-like protein. Scale bar = 20 μm. The black triangle represents the N-terminal of LbGAL4-like protein. (N) Colocalization study in *N. benthamiana* cells showing PtSSP1-GFP and LbGAL4-like-RFP colocalized in the nucleus, as analyzed by confocal microscopy. Scale bar = 20 μm. Note: Significant groups are indicated by a and b (*P* < 0.05).

**Figure 1:**
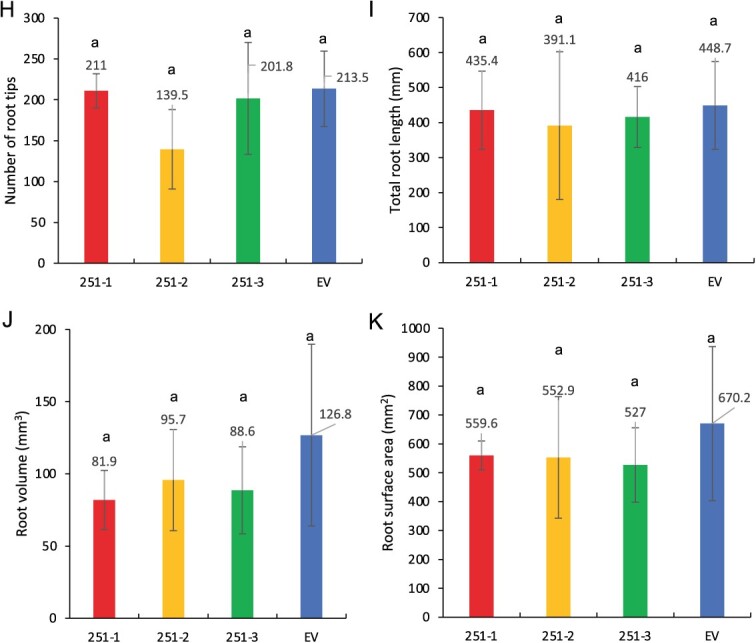
Continued.

**Figure 1:**
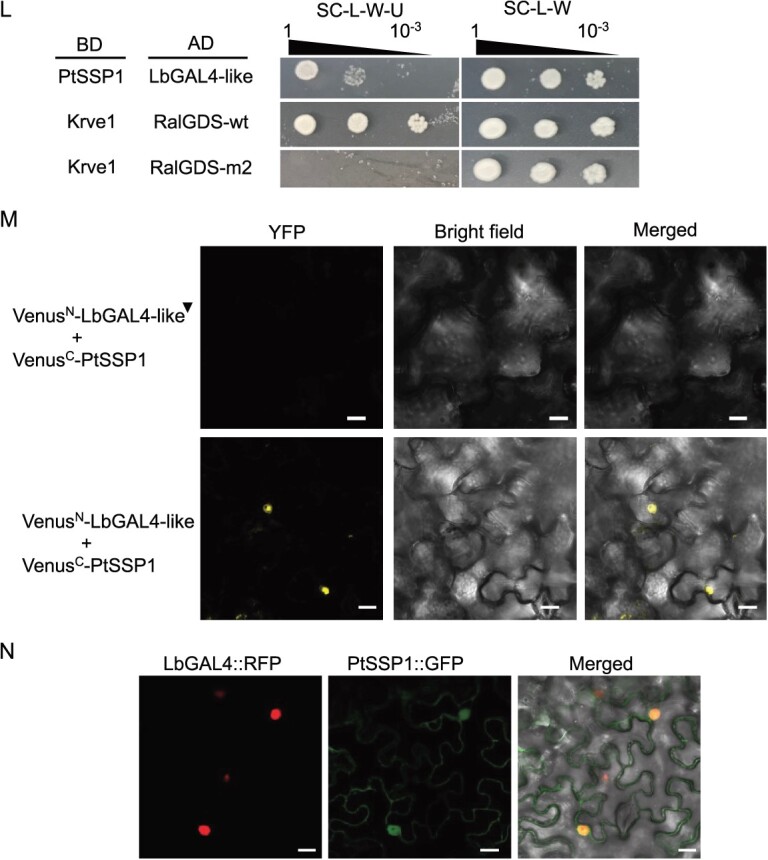
Continued.

To uncover additional potential effects of PtSSP1, we conducted a more comprehensive analysis on a subset of three to six ECM root tips per line using confocal microscopy to assess external (mantle) and *in planta* (Hartig net) fungal growth. Using a vibratome, we generated ten 30-μm cross-sections from each root tip and stained the samples using an established protocol [8]. After confocal imaging of complete cross-sections, we assessed four parameters: mantle width, root diameter, Hartig net boundary, and root circumference (Fig. 1E). These measurements were then used to calculate the ratio of mantle width to root diameter (as depicted in Fig. 1F) and the ratio of Hartig net boundary to root circumference (as illustrated in Fig. 1G). Compared with empty vector controls, in three lines, overexpression of *PtSSP1* caused from 6% to 19.5% and from 13% to 29.9% decrease in both ratios, respectively, among which two lines (251–1, 251–3) showed significant differences (*P* < 0.05, Fig. 1F and G). These results suggest that while overexpression of *PtSSP1* in *Populus* increase the number of ECM root tips, it also inhibits *in planta* fungal growth by *L. bicolor*, which is consistent with our previous discovery that the growth rate of *L. bicolor* hyphae was significantly reduced when treated with synthesized PtSSP1 [6].

It has been shown that plant SSPs also regulate plant development [9, 10]. To evaluate whether PtSSP1’s effect on mycorrhization is linked to a modification of root development, we measured different plant root parameters, including total root length, number of root tips, root volume, and root surface area. We found no significant difference in these root parameters between these overexpression lines and the empty vector controls (Fig. 1H–K), suggesting that PtSSP1 only functions during *L. bicolor* ectomycorrhization and likely by controlling *L. bicolor* development.

Previously, we have demonstrated that PtSSP1 can move across the hyphal membrane of *L. bicolor* and localize to the fungal nucleolus [6]. Hence, we hypothesized that PtSSP1 interacts with nuclear-localized fungal proteins to regulate *L. bicolor* ectomycorrhization in *Populus*. To test our hypothesis, we screened a yeast two hybrid (Y2H) library to identify the PtSSP1 partners in *L. bicolor*. Through the Y2H library screening, we found that PtSSP1 interacted with a GAL4-like transcription factor (TF) of *L. bicolor*, named LbGAL4-like (ID# 666247) (Fig. 1L). We further validated the interaction between PtSSP1 and LbGAL4-like using bimolecular fluorescence complementation (BiFC) (Fig. 1M). The protein colocalization assay in tobacco leaves showed that PtSSP1 displayed a nucleocytoplasmic localization, whereas LbGAL4-like only localized in the nucleus (Fig. 1N). These results suggest that PtSSP1 interacts with a fungal transcription factor, LbGAL4-like, to regulate the symbiosis between poplar and *L. bicolor.*

In conclusion, we demonstrated that PtSSP1 serves as an effector controlling symbiosis between *L. bicolor* and *Populus*, without affecting the root development. Furthermore, in *L. bicolor*, we identified a GAL4-like TF, LbGAL4-like, which can interact with PtSSP1. These results suggest that PtSSP1 interacts with TFs to control gene expression in *L. bicolor* and hereby regulate the ECM development. Further experiments are needed to fully reveal how PtSSP1-LbGAL4-like interaction changes the transcriptomic profile in *L. bicolor* and thereby controls the ECM development.

## Data Availability

All data supporting this study are available in the article.
